# *Proteomes* Annual Report Card 2025

**DOI:** 10.3390/proteomes14020022

**Published:** 2026-04-24

**Authors:** Jens R. Coorssen, Matthew P. Padula

**Affiliations:** 1 Institute for Globally Distributed Open Research and Education (IGDORE), St. Catharines, ON L2M 4X2, Canada; 2 Proteomics and Metabolomics Core Facility and School of Life Sciences, Faculty of Science, University of Technology Sydney, Sydney, NSW 2007, Australia

We begin by expressing our sincere thanks to all Editorial Board Members, Guest Editors, Reviewers, Authors, and the staff in the Editorial Office for their dedicated service in support of *Proteomes*. When we were asked to serve jointly as Editors-in-Chief, we had a vision to refocus the journal in a future-forward and future-proofing manner. Primarily, this involved recognising proteoforms as the actual constituents of proteomes, rather than continuing the ~25+ year focus on canonical amino acid sequences as a derivative of the Human Genome Project. In addition, we have also introduced criteria to ensure the rigour of publications in *Proteomes*. It is clear from the 2024 and 2025 data that, together with the committed work of our Editorial Board Members and reviewers, these changes are being widely recognized as original and meaningful by both authors and scientific organizations. Indeed, we actively seek engaging submissions from researchers working across the full spectrum of proteomics. We also see strong potential for further growth in areas such as bioinformatics, instrumentation, and methods development.

We would also like to note that over the last year, we have introduced criteria for the proposal/planning of Special Issues in a targeted effort to provide focus on subjects of high-interest or of critical ongoing development and/or application. In this regard, and as we had hoped with the targeted, revised focus and criteria at *Proteomes*, there has been a significant increase in the number of primary research papers published; correspondingly, we feel that the ~20% level of contribution, each, from Special Issues and review articles is appropriate for the direction of the journal in terms of growth, focus, and recognition. Furthermore, introducing more stringent expectations for review articles to be critical and integrative in their handling of data reported in the field, as well as providing insight into what is working or not—in order to highlight what is needed to move proteomics research forward—has yielded more consistency in the quality and rigour of submissions; reviews that simply regurgitate what papers in the field have said are no longer considered appropriate for publication in *Proteomes*. This has also led to the introduction of Perspective articles that enable experienced researchers to highlight current issues and/or developments in proteomics, with emphasis on impact in terms of future directions; again, these are grounded in a balanced, transparent, and critical evaluation of the existing literature (particularly within the last ~3 years) rather than functioning as a soapbox for the promotion of any particular methodological approach, organization, or institution.

In 2025, *Proteomes* made significant progress in the following areas:

- *Proteomes* has now published 13 volumes, with 48% of articles cited 10 times or more.
- The total number of article views increased by 13% to 549,674 as compared to 2024.
- We saw an increase in submissions of 73% and maintained our rejection rate from 2024.
- The journal featured contributions from 436 authors across 35 countries and regions, and our peer review was supported by 158 reviewers from 41 countries and regions.
- The median publication time (days from submission to publication) was 76 days, and a first decision was provided to authors approximately 29 days after submission.
- We received an Impact Factor of 3.6, released in the Journal Citation Report 2024 (Clarivate Analytics), and ranked 129/320 (Q2) in the category of “Biochemistry & Molecular Biology”.
- We received an increased CiteScore (Scopus) of 7.2 in 2024, ranking Q1 in both the “Structural Biology” and “Clinical Biochemistry” categories.
- We engaged with our scientific community at several conferences, including the Swiss Proteomics Meeting 2025 and HUPO 2025 (Table 1).
- We announced the journal’s first Outstanding Reviewer Award and reintroduced the Best Paper Award after a three-year hiatus.

With increases in so many critical journal evaluation criteria (e.g., publications, readership, subscriptions, international engagement), it is clear that *Proteomes* has established itself as a recognized, trusted, and approachable journal despite now entering only its 13th year of publication. We anticipate these results will translate into another solid ranking of the journal for the 2025 reporting year. The field is going through a period of technical changes, critical (re-)evaluation, and a long-overdue recognition of the actual complexity of proteomes and thus of the approaches needed to ensure transparency and rigour of analyses, from the moment of sample collection through to final statistical analyses and data presentation. *Proteomes* is well-positioned as a key partner in ensuring and promoting these necessary ongoing and future directions.

Lastly, we appeal to submitting authors and Editorial Board Members handling new submissions to ensure that submitted manuscripts adhere to all the journal’s guidelines, that methodologies are thoroughly presented and thus enable the study to be fully reproduced by other researchers, that results are properly and thoroughly presented, and that interpretations are made with a clear recognition, if not full focus, on proteoforms. Non-conforming manuscripts will be returned to authors for revision so that reviewers’ valuable time can be spent assessing the quality of the science.

## Figures and Tables

**Table 1:** Engagements with the scientific community in 2025.

Conference	Sponsorship Title	Date	Location	Photo
Swiss Proteomics Meeting 2025	Best Oral Award	15–16 May 2025	Unterägeri, Switzerland	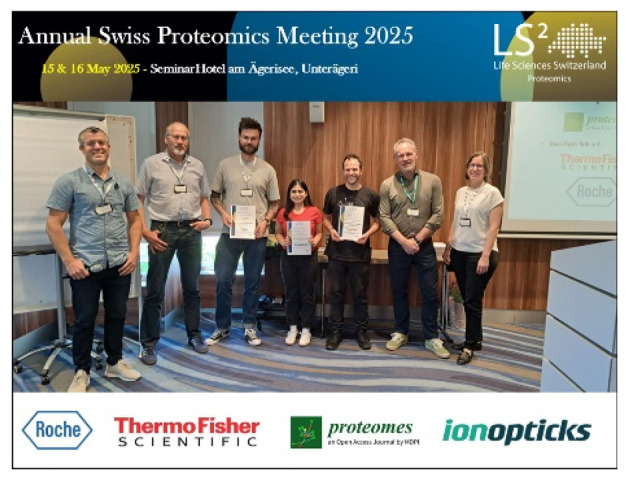
HUPO 2025	Journal Poster	9–13 November 2025	Toronto, Canada	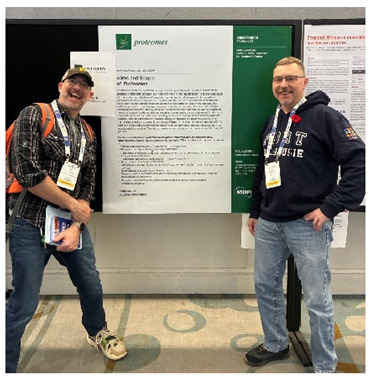 Left: Dr. Benjamin Orsburn (one of our Editorial Board Members); Right Prof. Dr. Jens R. Coorssen (joint Editor-in-Chief)

